# Effect of adjunctive lidocaine-based scalp block and laryngotracheal local anesthesia vs general anesthesia only on plasma and cerebrospinal fluid pro-inflammatory cytokine concentrations in patients with cerebral aneurysm**: a** randomized controlled trial

**DOI:** 10.3325/cmj.2021.62.338

**Published:** 2021-08

**Authors:** Marijana Matas, Vlatka Sotošek, Ana Kozmar, Robert Likić, Goran Mrak, Bálint Nagy, Ante Sekulić

**Affiliations:** 1 Department of Anesthesiology, Reanimatology and Intensive Care Medicine, University Hospital Center Zagreb, Zagreb, Croatia; 2 Department of Anesthesiology, Resuscitation, Emergency and Intensive Care Medicine, Faculty of Medicine, University of Rijeka, Rijeka, Croatia; 3 Department of Laboratory Diagnostics, University Hospital Center Zagreb, Zagreb, Croatia; 4 Department of Internal Medicine, Unit of Clinical Pharmacology, University Hospital Center Zagreb, Zagreb, Croatia; 5 Department of Neurosurgery, University Hospital Center Zagreb, Zagreb University School of Medicine, Zagreb, Croatia; 6 Department of Anesthesiology and Intensive Therapy, Medical School, University of Pécs, Pécs, Hungary

## Abstract

**Aim:**

To compare the effect of adjunctive lidocaine-based scalp block and laryngotracheal local anesthesia vs general anesthesia only on pro-inflammatory cytokine concentrations in patients with non-ruptured brain aneurysms undergoing elective open surgery.

**Methods:**

This parallel, randomized, controlled, open-label trial was conducted at Clinical Hospital Center Zagreb between March 2019 and March 2020. At the beginning of anesthesia, lidocaine group received 40 mg of 2% lidocaine for laryngotracheal topical anesthesia and 4 mg/kg for the scalp block. Control group underwent general anesthesia only. Plasma concentrations of IL-6, TNF-α, and IL-1β were measured before anesthesia (S0); at the incision (S1); at the end of surgery (S2); 24 hours postoperatively (S3). Cerebrospinal fluid (CSF) cytokine concentrations were measured at the incision (L1) and the end of surgery (L2).

**Results:**

Forty patients (each group, 20) were randomized; 37 were left in the final analysis. IL-6 plasma concentrations increased significantly compared with baseline at S3 in lidocaine group, and at S2 and S3 in control group. In both groups, changes in TNF-α and IL-1β were not significant. CSF cytokine concentrations in lidocaine group did not change significantly; in control group IL-6 and IL-1β were significantly higher at L2 than at L1. CSF IL-6 in control group significantly increased at L2, but TNF-α and IL-1β did not. No differences in clinical outcome and complication rates were observed.

**Conclusion:**

Adjunctive lidocaine-based scalp block and laryngotracheal local anesthesia might attenuate CSF IL-6 concentration increase in patients with brain aneurysm.

**Trial registration:**

Clinical Trials NCT03823482

The prevalence of cerebral aneurysms in adults is about 2.8% (1). Unruptured aneurysms are usually asymptomatic, but a wider availability of neuroimaging increased their incidental detection. Patients with cerebral aneurysms usually undergo endovascular or open surgery, both of which are burdened by significant disability and mortality (2). Although the pathophysiological mechanism of cerebral aneurysms is not fully understood, recent research has shown that inflammation plays a crucial role in its origin and complications, such as rupture or hemorrhage (3).

Brain tissue injury triggers an inflammatory response and a release of various inflammatory mediators, such as cytokines, which leads to secondary brain injury. Increased concentrations of pro-inflammatory cytokines and extracellular adenosine triphosphate (ATP) were found in plasma and cerebrospinal fluid (CSF) during and after craniotomy, as well as after cerebral aneurysm rupture (4-6). This excessive release of pro-inflammatory mediators activates microglia. Microglia additionally secrete interleukin-1β (IL-1β), interleukin-6 (IL-6), and tumor necrosis factor-α (TNF-α), which migrate to systemic circulation through a damaged blood brain barrier (BBB) (7).

Earlier studies have shown that elevated IL-6 and TNF-α concentrations in plasma and CSF in patients with aneurysmatic subarachnoid hemorrhage (SAH) correlate with the incidence of complications and overall outcome (8,9).

Lidocaine is a local anesthetic that exerts significant neuroprotective effect, has anti-inflammatory properties, and reduces metastasis occurrence (10). For anesthesia in neurosurgery, lidocaine can be used before laryngoscopy and for scalp block to blunt the autonomic response, which leads to increased blood and intracranial pressure (11-14).

Anti-inflammatory properties of lidocaine have been extensively investigated in *in vitro* and *in vivo* models. Lidocaine exerts its anti-inflammatory effect through the modulation of pro-inflammatory mediator release by lymphocytes (15-17). Although not equivocally, clinical studies have shown that intravenously and locally administered lidocaine might reduce pro-inflammatory cytokine concentrations (18,19). Additionally, in the central nervous system (CNS), lidocaine reduces IL-1β release, thus attenuating microglial activities, which are considered crucial for the development of secondary brain injury (20).

The impact of lidocaine on inflammatory response during and after cerebral aneurysm surgery has rarely been investigated. A recent study found that scalp block attenuated the inflammatory response after craniotomy, although cytokine concentrations were measured in plasma only (21). The impact of adjunctive lidocaine-based scalp block and laryngotracheal local anesthesia on plasma and CSF cytokine concentrations during and after cerebral aneurysm surgery has not been investigated so far. Therefore, we hypothesized that lidocaine applied for scalp block and topically for laryngotracheal anesthesia prevented pro-inflammatory cytokines release in CSF and plasma in patients with unruptured brain aneurysms during and after surgery. The primary aim was to determine the effect of adjunctive regional/topical lidocaine anesthesia on perioperative temporal profile of pro-inflammatory cytokines concentrations (IL-1β, IL-6, and TNF-α) in plasma and CSF of cerebral aneurysm patients undergoing elective open surgery. The secondary aim was to evaluate the influence of adjunctive lidocaine on the incidence of complications and outcome.

## PATIENTS AND METHODS

### Study design

This randomized, single-center, open-label, controlled clinical trial was conducted at University Hospital Center Zagreb. Patients were recruited from March 2019 until March 2020. The study protocol was performed according to the ethical guidelines outlined in the Declaration of Helsinki and was entirely published earlier (22). Ethical approval was obtained from the Institutional Review Board of the University Hospital Center Zagreb, Zagreb (02/21 AG) and Ethics Committee of Faculty of Medicine, University of Rijeka, Rijeka (217 0-24-04-3-19-3).

### Inclusion criteria

Forty patients with cerebral aneurysm scheduled for elective open surgery were consecutively included in the study. All signed informed consent. We did not enroll patients if they 1) had severe cardiovascular or pulmonary condition, 2) had acute infection, 3) received steroids, 4) had preoperative Glasgow coma score (GCS)<15, 5) had a history of allergy to lidocaine, 6) were >70 or <18 years old .

**Figure 1:**
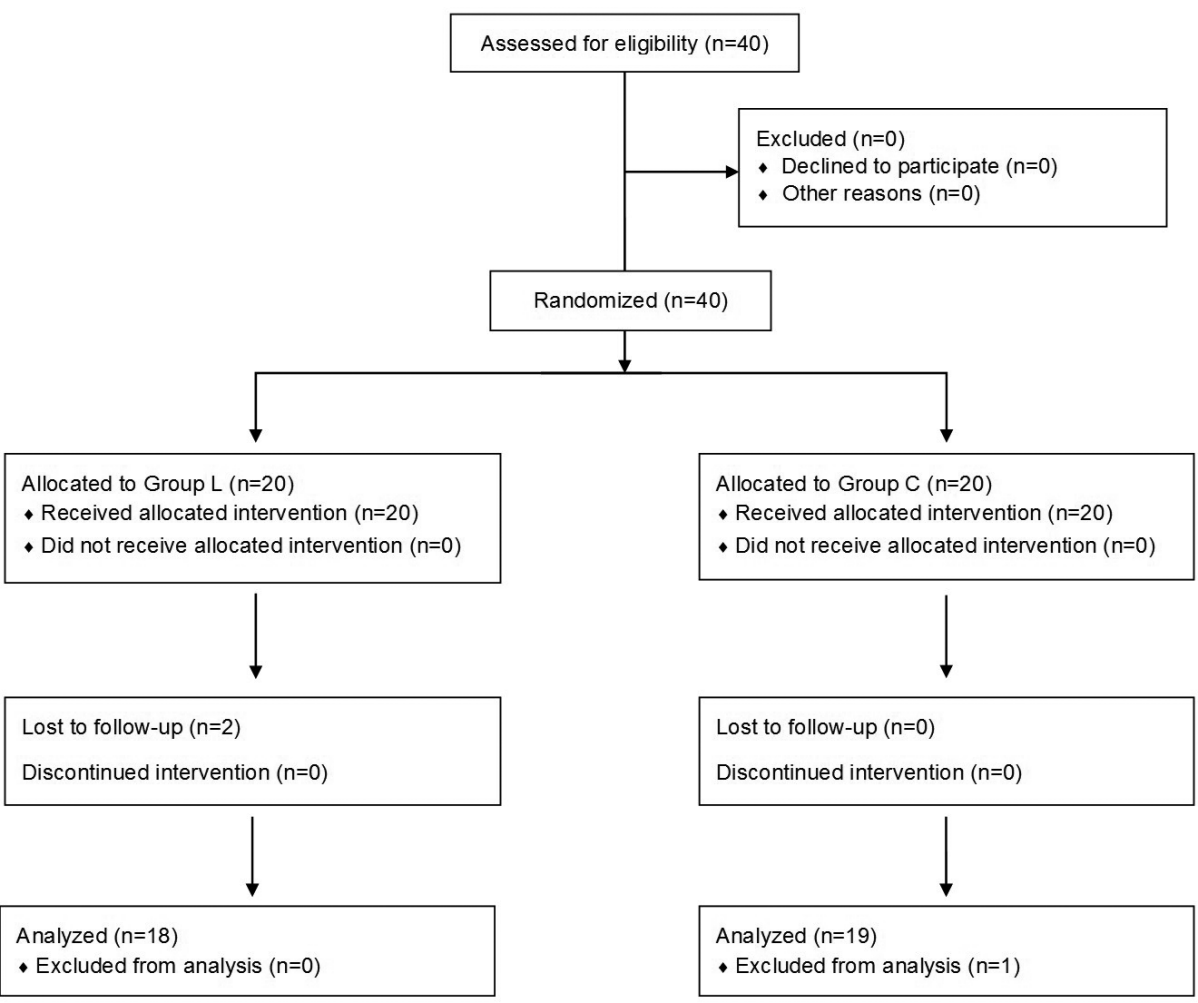
Study flow diagram. Abbreviations: L – lidocaine; C – control.

Patients were randomized before the surgery into the control or lidocaine group with a computer-based 1:1 random allocation. Two patients in the lidocaine group were lost to follow-up, while one control patient was excluded due to significant intraoperative bleeding (Figure 1).

### Intervention

The patients allocated to the lidocaine group received general anesthesia. Before laryngoscopy and endotracheal intubation, 40 mg of lidocaine (Lidokain Belupo, Koprivnica, Croatia) was applied topically to the throat. Additionally, the patients in this group received scalp nerves block, which was performed with 2% lidocaine in a dose of 4 mg/kg. The full amount of lidocaine did not exceed 400 mg. Scalp block was applied using an established, previously described technique (23). The patients allocated to the control group received general anesthesia only.

### Anesthesia and surgical procedure

All patients underwent craniotomy and aneurysm clipping. Lumbar CSF drainage was placed preoperatively when it was neurosurgically indicated. Standard intraoperative monitoring was used: electrocardiography, pulse oximetry, invasive and noninvasive blood pressure measurement, capnography, body temperature, and urine output. A catheter was placed in the radial artery to continuously monitor arterial blood pressure. GE-Entropy Module (GE Healthcare, Helsinki, Finland) was used to monitor the processed EEG variables, response entropy (RE) and state entropy (SE), for the purpose of assessing the depth of anesthesia and adjusting the doses of anesthetic drugs to maintain RE/SE values between 40-60. General anesthesia was induced with fentanyl (Fentanyl Janssen Janssen Pharmaceutica, Beerse, Belgium), propofol (Propofol-Lipuro 20 mg/mL MCT Fresenius, Fresenius Kabi, Graz, Austria), and rocuronium (Esmeron, Merck Sharp & Dohme, Kenilworth, NJ, USA). Following tracheal intubation, patients were mechanically ventilated. Anesthesia was maintained by propofol infusion at a dose of 100–200 µg/kg/min and fentanyl at a dose of 1-2 µg/kg/h. Any changes of arterial blood pressure of ±30% of baseline values were corrected by adjusting the dose of propofol, adding bolus of fentanyl and/or vasoactive drugs (ephedrine, phenylephrine, noradrenaline).

### Postoperative complications and outcome measurement

Patients were monitored for complications until discharge. Significant neurological complications were new focal motor deficit, decrease in GCS by ≥2 points, seizures, vasospasm, and other pathological findings on brain CT scan (bleeding, ischemia, edema, or hydrocephalus). Infectious complications included meningitis, pneumonia, and sepsis.

Motor deficit and seizure were assessed by clinical examinations. Vasospasm was diagnosed by transcranial ultrasound. Meningitis, pneumonia, and sepsis were diagnosed according to the established diagnostic criteria (24-26). Glasgow outcome scale (GOS) was recorded at discharge (27).

### Sampling and biochemical analysis

Blood samples (16 mL of blood in total) were collected via arterial line at four time points: S0 – before anesthesia; S1 – at the incision; S2 – at the end of surgery; and S3 - 24 hours after surgery. CSF samples (4 mL of CSF in total) were drawn via the lumbar CSF drainage system at two time points: L1 – at the incision and L2 – at the end of surgery. Since lumbar drainage was not placed in all patients, CSF samples were collected, processed, and analyzed in only seven patients from the lidocaine group, and nine patients from the control group.

Plasma from arterial blood and CSF samples was obtained by centrifugation and stored at -80 °C until analysis. IL-1β, L-6, and TNF-α concentrations were measured by enzyme-linked immunosorbent assay according to the manufacturer's instructions (BioVendor L.M., A.S., Brno, Czech Republic) and assessed spectrophotometrically (Sunrise microplate photometer, Tecan Trading AG, Männedorf, Switzerland), by reading absorbance at 450 nm. The concentrations were expressed as picogram/milliliter (pg/mL).

### Statistical analysis

Continuous data are presented as median and interquartile range (IQR), and categorical data as absolute and relative frequencies. Distribution normality was tested with the Shapiro-Wilk or Kolmogorov-Smirnov test. Friedman’s two-way ANOVA with post-hoc Dunn’s test, Mann-Whitney *U* test, Wilcoxon test, and Fischer`s test were used to assess the within and between-group differences. The level of significance was set at *P* < 0.05. The analysis was performed with SPSS 25.0 (IBM Corp., Armonk, NY, USA).

## RESULTS

The groups did not differ in any of the demographic variables, except age. Patients in the lidocaine group were significantly younger than controls (54 ± 8 vs 59 ± 10, *P* = 0.01). The groups did not significantly differ in any of the anesthesia- and surgery-related variables, including total duration of anesthesia, total duration of surgery, and total dosage of propofol and fentanyl, as well as the number of patients needing vasopressors (Table 1).

**Table 1:** Demographic and perioperative characteristics of the lidocaine group and control group patients*^†^

	Lidocaine group (n = 18)	Control group (n = 19)	*P*
Age (years)	54 ± 8	59 ± 10	0.010
Sex (male/female)	5/13	2/17	0.180
BMI (kg)	25.5 ± 4.8	25.6 ± 4.8	0.815
ASA status (ASA I/ASA II)	3/15	3/16	0.643
Aneurysm location (ACI/ACM/AcoA/AcoP)	3/13/2/0	2/12/3/2	0.407
Multiple aneurysm (yes/no)	10/8	9/10	0.433
Hypertension as coexisting disease (yes/no)	10/8	14/5	0.209
Diabetes as coexisting disease (yes/no)	0/18	0/19	0.209
Total duration of operation including anesthesia (min)	245 (93)	270 (130)	0.753
Duration of surgery without anesthesia time (min)	185 (83)	200 (103)	0.443
Urine output (mL)	300 (225)	400 (588)	0.374
Total dose of propofol (mg)	1760 (518)	1950 (1240)	0.461
Total dose of fentanyl (mg)	0.8 (0.15)	0.8 (0.3)	0.940
Total dose of lidocaine (mg)	255 (33)	0 (0)	N/A
Total dose of intraoperative crystalloid infusion (mL)	2500 (625)	2250 (500)	0.480
Need for intraoperative vasopressor (yes/no)	4/14	8/11	0.174

*Abbreviations: ACI – arteria carotis interna; ACoA – arteria communicans anterior; ACoP – arteria communicans posterior; ACM – arteria cerebri media; ASA – American Society of Anesthesiology; BMI – body mass index; N/A – non-applicable; SD – standard deviation.

†Numbers are median (interquartile range), mean ± standard deviation, or absolute frequencies.

### Perioperative plasma dynamics of IL-6, TNF-α and IL-1β

**Figure 2:**
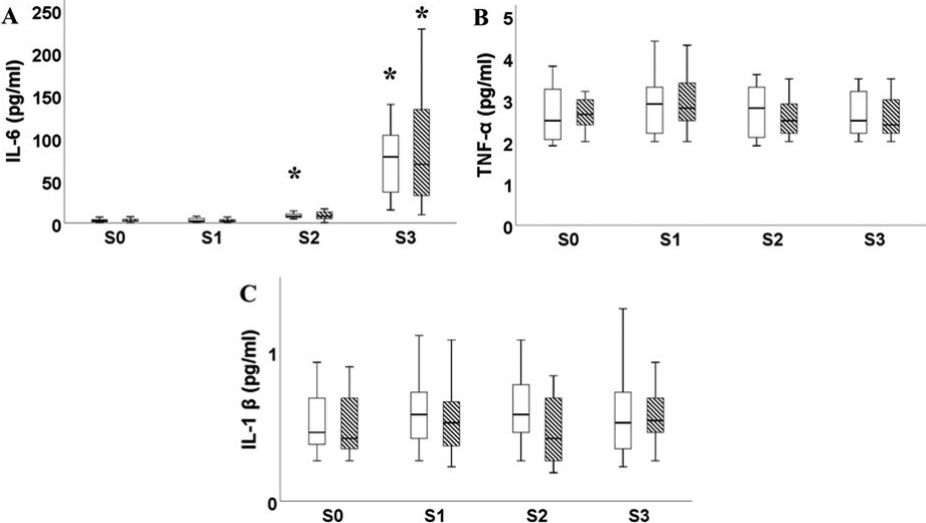
Plasma concentrations of interleukin-6 (IL-6) (**A**), tumor necrosis factor-α (TNF-α) (**B**), and interleukin-1β (IL-1β) (**C**) at four different time points (S0: before induction of anesthesia; S1: incision; S2: end of surgery; S3: 24 hours postoperatively). Data are expressed as minimum, maximum, median, and interquartile range. White boxes represent control patients; shaded boxes show lidocaine group results. Asterisk indicates significant differences (*P* < 0.05) within the groups compared with S0.

In the control group, IL-6 concentrations in plasma were significantly higher at S2 (*P* = 0.032) and S3 when compared with S0 (*P* < 0.001) (Figure 2A). TNF-α (Figure 2B) and IL-1β concentrations (Figure 2C) in plasma did not change at any time point. In the lidocaine group, IL-6 concentrations were higher at S3 compared with S0 (*P* < 0.001) (Figure 2A); TNF-α (Figure 2B) and IL-1β (Figure 2C) concentrations did not change at any time point.

### Changes of concentrations of IL-6, TNF-α, IL-1β in cerebrospinal fluid

**Figure 3:**
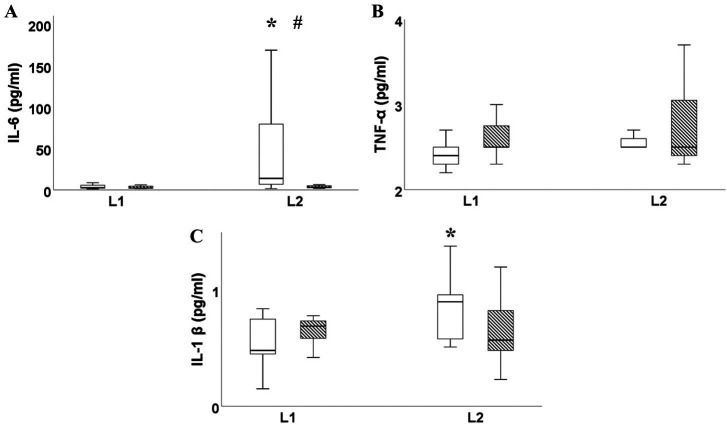
Cerebrospinal fluid (CSF) concentrations of interleukin-6 (IL-6) (**A**), tumor necrosis factor-α (TNF-α) (**B**), and interleukin-1β (IL-1β) (**C**) at two time points (L1: time of skin incision; L2: end of surgery). Data are expressed as minimum, maximum, median, and interquartile range. White boxes represent control patients; shaded boxes show lidocaine group results. Asterisk indicates significant differences (*P* < 0.05) within the groups compared with L1, while octothorp indicates significant differences (*P* < 0.05) between the groups.

In the control group, CSF concentrations of IL-6 (Figure 3A) (*P* = 0.008) and IL-1β (Figure 3C) were significantly higher at L2 compared with L1 (*P* = 0.012). TNF-α concentrations did not change significantly (Figure 3B). In the lidocaine group, IL-6 (Figure 3A), TNF-α (Figure 3B), and IL-1β (Figure 3C) concentrations in CSF did not change significantly.

CSF IL-6 concentrations at L2 were significantly higher in the control group compared with the lidocaine group (*P* = 0.042) (Figure 3A), but this was not the case for TNF-α (Figure 3B) and IL-1β (Figure 3C).

### Incidence of neurological and infectious complications

The most frequent complications in both groups were decrease in GCS and new neurological deficits due to ischemia. The incidence of neurological complications did not differ between the groups (Table 2). The most frequent infectious complication was meningitis. Other complications and laboratory test results did not differ between the groups (Table 3).

**Table 2:** Neurological complications in the lidocaine group and control group patients*^†^

	Lidocaine group (n = 18)	Control group (n = 19)	*P*
Decrease in GCS≥2 (yes/no)	2/16	5/14	0.225
New neurological deficit (yes/no)	2/16	5/14	0.225
Vasospasm proven by TCD/angio/clinical signs (yes/no)	2/16	4/15	0.357
Ischemia/stroke proven by CT (yes/no)	2/16	5/14	0.225
Seizure (yes/no)	2/16	2/17	0.677
Postoperative bleeding proven by CT (yes/no)	1/17	3/16	0.323
Reoperation (yes/no)	1/17	2/17	0.521
Brain edema proven by CT (yes/no)	0/18	0/19	N/A
Hydrocephalus proven by CT (yes/no)	0/18	0/19	N/A

*Abbreviations: CT – computed tomography; GCS – Glasgow Coma Score; N/A – non-applicable; TCD – transcranial Doppler.

†Numbers are frequencies.

**Table 3:** Infectious complications and related laboratory parameters in the lidocaine group and control group patients*^†^

	Lidocaine group (n = 18)	Control group (n = 19)	*P*
Meningitis (yes/no)	3/15	5/14	0.379
Pneumonia (yes/no)	1/17	4/15	0.187
Sepsis (yes/no)	1/17	1/18	0.743
White blood cell count (first postop day) ( × 10^9^/L)	6.8 (3.9)	6.4 (3.3)	0.620
White blood cell count (third postop day) ( × 10^9^/L)	10.2 (3)	9.8 (4.1)	0.372
CRP (first postop day) (mg/L)	0.75 (1.3)	0.8 (1.1)	0.940
CRP (third postop day) (mg/L)	48.5 (83)	62.3 (71.2)	0.461

*Abbreviations: CRP – C-reactive protein.

†Numbers are median (interquartile range) or frequencies.

### Treatment outcome

GOS<5 was observed in two lidocaine group patients (11%), while 89% had maximum scores (GOS = 5). In control group, 26% of the patients had GOS<5 at discharge.

## DISCUSSION

The results of this study show that elective cerebral aneurysm surgery is followed by increased IL-6 concentrations in plasma and increased IL-6 and IL-1β concentrations in CSF. Lidocaine administration mitigated the increase in CSF IL-6 concentration, which might suggest an effect on local inflammatory response. Nevertheless, we were unable to show a significant difference in postoperative neurologic and infectious complications and treatment outcome.

Craniotomy is associated with a postoperative inflammatory response via a pro-inflammatory cytokine release (4,5,7,28). Our results on perioperative plasma IL-1β, IL-6, and TNF-α dynamics agree with previous research. In elective craniotomy patients, Heesen et al found peak plasma IL-6 concentrations 24 hours postoperatively (4). Other studies also showed similar plasma IL-6 elevation, with peak concentrations at 24 hours postoperatively (5,21).

We noted a transient, non-significant elevation of plasma IL-1β and TNF-α concentrations at the beginning of surgery, which returned to baseline by the end of surgery. This is in line with the study by Osuka et al (28).

Only a few studies report on perioperative CSF cytokine concentrations in craniotomy patients. Woiciechowsky et al (6) found a significant IL-6 increase in CSF 2-4 hours after craniotomy, followed by a decline. Similarly, we observed a significant increase in IL-6 CSF concentration in the control group and a non-significant increase in the lidocaine group. Woiciechowsky et al (6) also found increased CSF TNF-α concentrations and no change in CSF IL-1β concentrations – these findings are all contrary to our results (6). However, Woiciechowsky et al studied intra-axial tumor patients, who have more extensive surgical trauma, and used an intraventricular drain to draw CSF samples, which also might have affected the cytokine profile (29). The elevation of IL-1β, IL-6, and TNF-α concentrations in CSF is also observed in patients with SAH (30,31). Interestingly, a recent study found a difference in the dynamics of intrathecal and systemic IL-6 concentrations in SAH patients, suggesting higher intensity of inflammatory process intrathecally, which might explain our results in this regard (30).

Local anesthetic administration showed promising results with regard to craniotomy-associated anti-inflammatory effects, but published data are inconclusive and further research is needed (14,15,21,32). In our study, lidocaine administration significantly diminished CSF IL-6 increase at the end of surgery. IL-1β and TNF-α CSF concentrations in the lidocaine group did not significantly differ. Overall, lidocaine affected the local but not the systemic inflammatory response. Contrary to our results, Yang et al (21) found significantly lower plasma IL-6 concentrations six hours after surgery in patients who received scalp block with ropivacaine, also supporting the potential transient systemic anti-inflammatory effect of the procedure. However, they did not observe significant differences in IL-6 concentrations in plasma 24 hours after craniotomy.

Cytokines, especially pro-inflammatory cytokines, might play a role in postoperative complications following cerebral aneurysm surgery (8). We found focal ischemia to be the most frequent neurological complication (18.9%), which agrees with recent data (33). The most frequent infectious complication was meningitis (21.6%). The relatively high meningitis incidence may be explained by lumbar CSF drainage. Chen at al found meningitis incidence to be up to 44.7% in patients with lumbar CSF drainage system (34). Even though the incidence of complications did not significantly differ between the groups, the overall occurrence of complications was lower in the lidocaine group, which may favor the safety profile of the drug in this indication.

Unfavorable treatment, described as GOS≤4 at hospital discharge, was reported in 13.5% and 27.6% cerebral aneurysm patients in a study by Thopmson et al (35). Similarly, we found GOS≤4 in 18.9% of our patients, without significant difference between the groups.

The study has several limitations. Our trial was single centered, which might have influenced the number of complications and the outcome. Despite randomization, patients in the control group were older than those in the lidocaine group. Exact plasma lidocaine concentrations were not measured, which might have influenced our results (17-19).

In conclusion, cerebral aneurysm surgery is accompanied by significant dynamic changes of pro-inflammatory mediators, such as cytokines. Lidocaine administration, both for topical laryngotracheal anesthesia and scalp nerve block, attenuates IL-6 increase in the CSF of patients undergoing elective craniotomy and aneurysm clipping. Our findings might confirm potential anti-inflammatory effects of lidocaine, support its usage in cerebrovascular surgery, and warrant further larger-scale studies.
